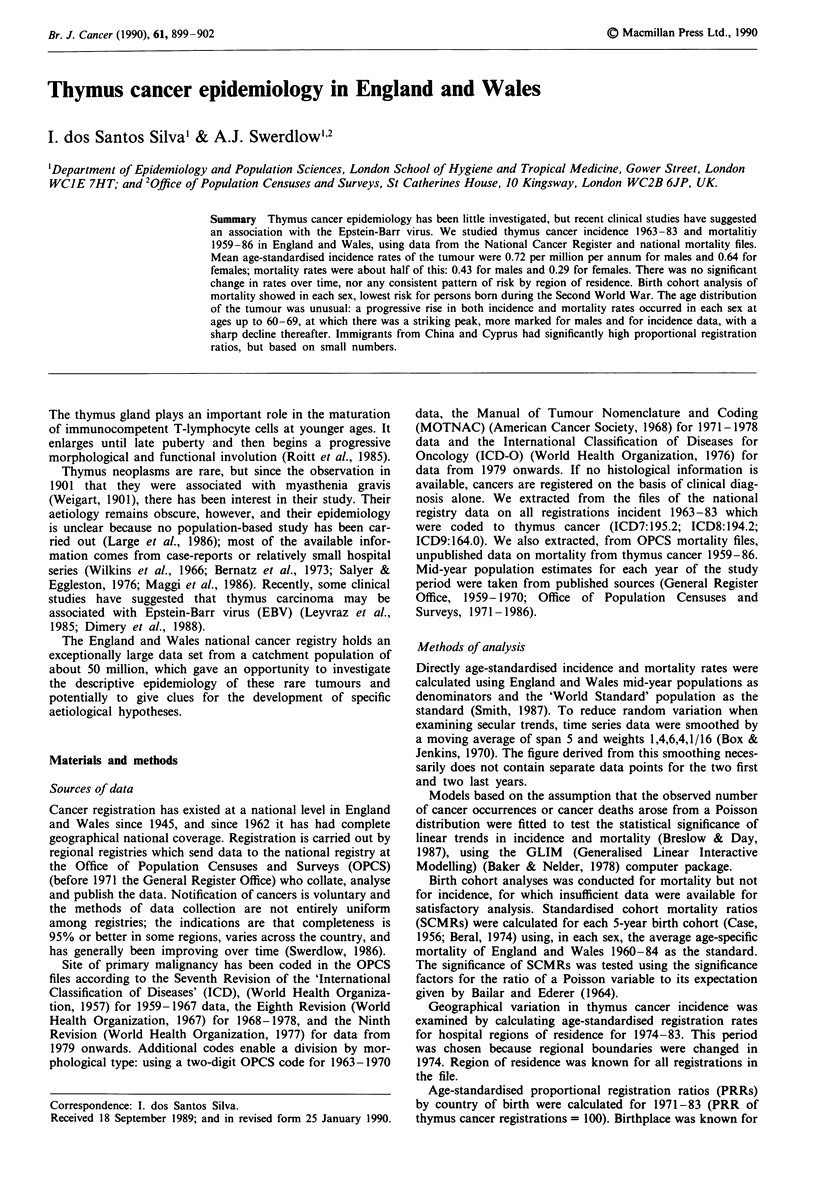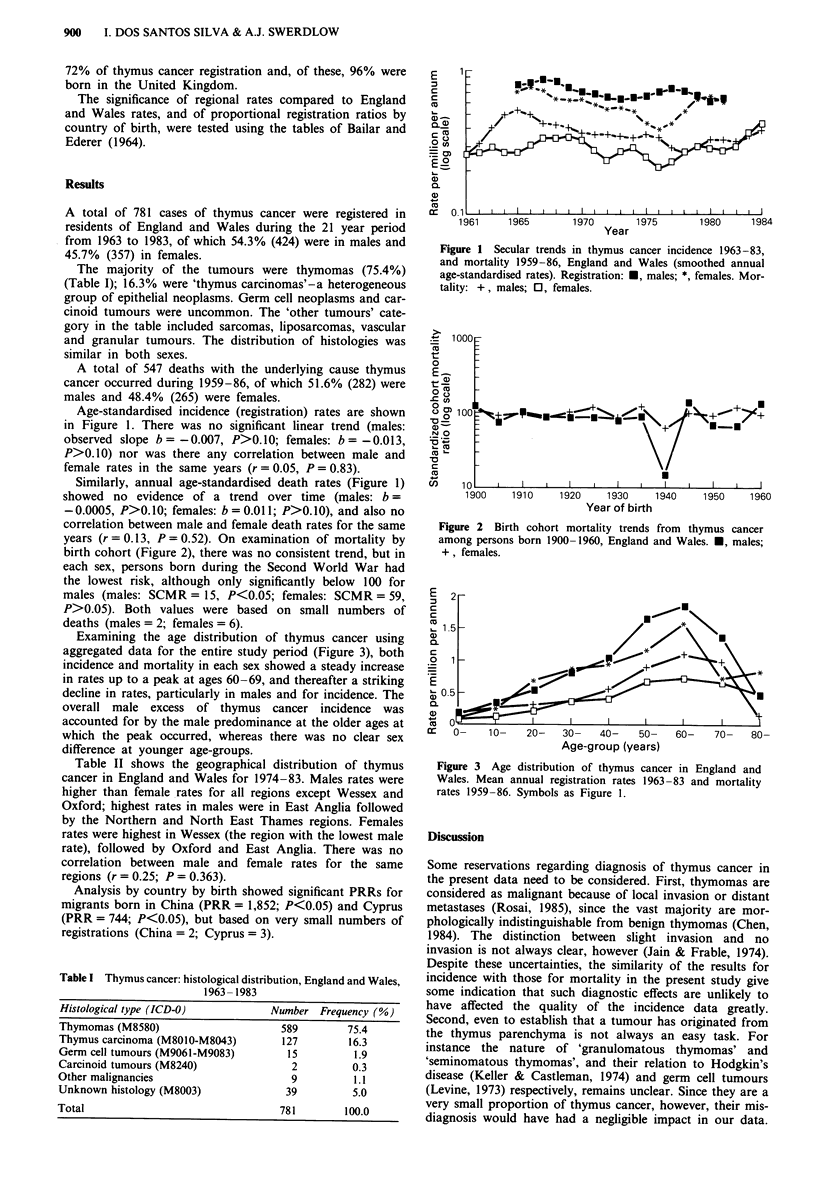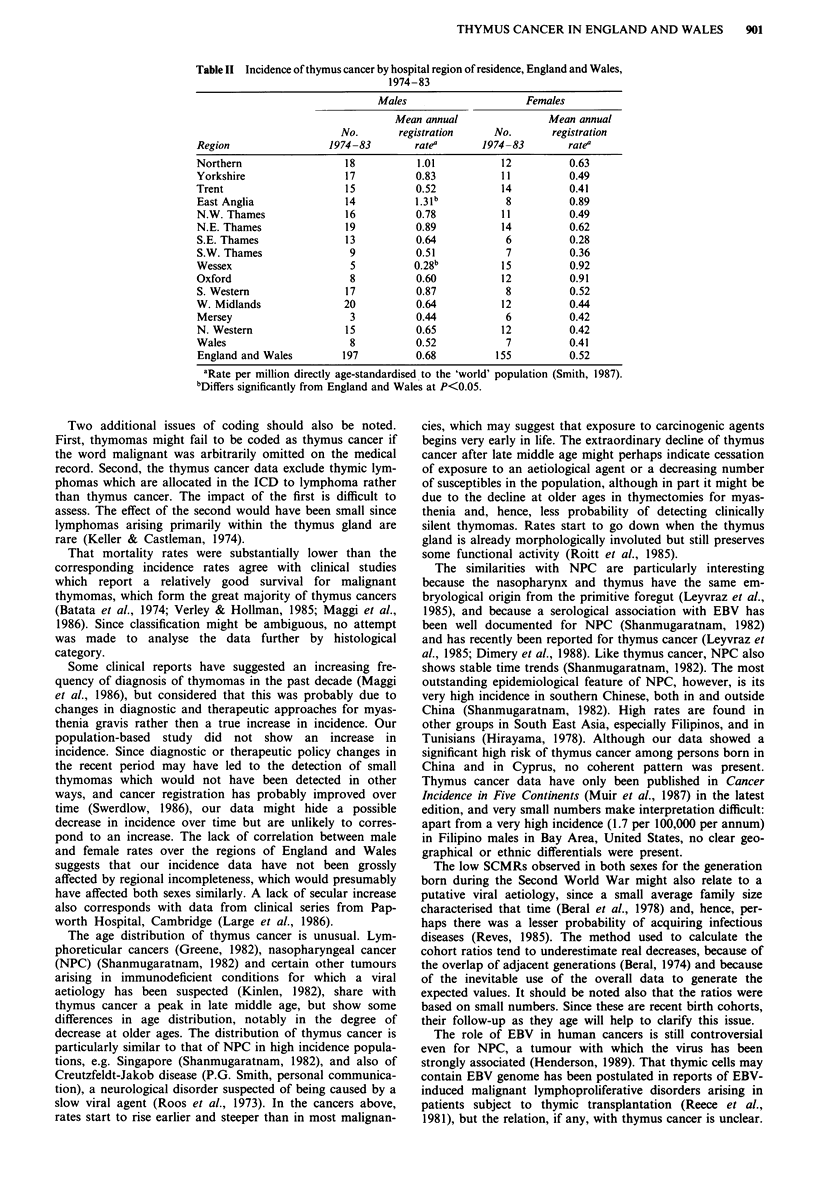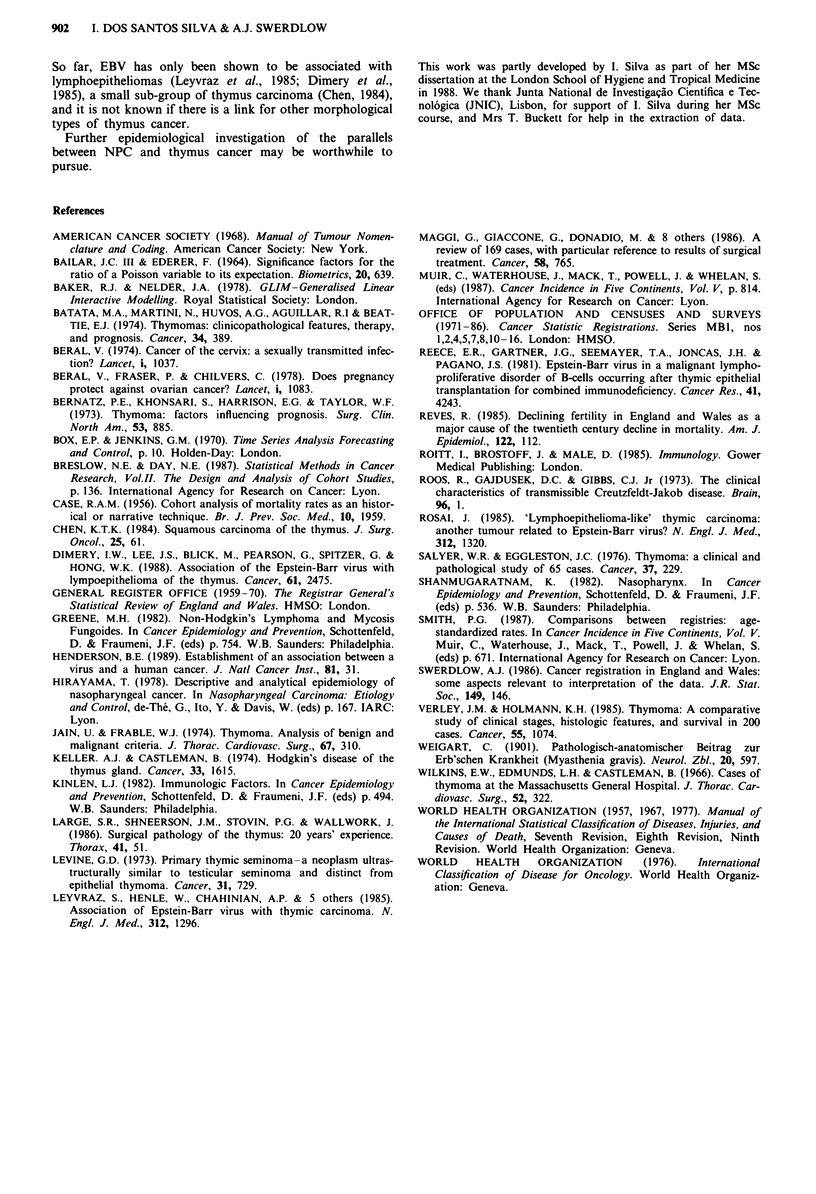# Thymus cancer epidemiology in England and Wales.

**DOI:** 10.1038/bjc.1990.201

**Published:** 1990-06

**Authors:** I. dos Santos Silva, A. J. Swerdlow

**Affiliations:** Department of Epidemiology and Population Sciences, London School of Hygiene and Tropical Medicine, U.K.

## Abstract

Thymus cancer epidemiology has been little investigated, but recent clinical studies have suggested an association with the Epstein-Barr virus. We studied thymus cancer incidence 1963-83 and mortality 1959-86 in England and Wales, using data from the National Cancer Register and national mortality files. Mean age-standardised incidence rates of the tumour were 0.72 per million per annum for males and 0.64 for females; mortality rates were about half of this: 0.43 for males and 0.29 for females. There was no significant change in rates over time, nor any consistent pattern of risk by region of residence. Birth cohort analysis of mortality showed in each sex, lowest risk for persons born during the Second World War. The age distribution of the tumour was unusual: a progressive rise in both incidence and mortality rates occurred in each sex at ages up to 60-69, at which there was a striking peak, more marked for males and for incidence data, with a sharp decline thereafter. Immigrants from China and Cyprus had significantly high proportional registration ratios, but based on small numbers.


Br. J. Cancer (1990), 61, 899-902 © Macmillan Press Ltd., 1990

### Thymus cancer epidemiology in England and Wales

I. dos Santos Silva[1] & A.J. Swerdlow[1,2]

[1]Department of Epidemiology and Population Sciences, London School of Hygiene and Tropical Medicine, Gower Street, London WC1E 7HT; and [2]Office of Population Censuses and Surveys, St Catherines House, 10 Kingsway, London WC2B 6JP, UK.

**Summary** Thymus cancer epidemiology has been little investigated, but recent clinical studies have suggested an association with the Epstein-Barr virus. We studied thymus cancer incidence 1963-83 and mortalitiy 1959-86 in England and Wales, using data from the National Cancer Register and national mortality files. Mean age-standardised incidence rates of the tumour were 0.72 per million per annum for males and 0.64 for females; mortality rates were about half of this: 0.43 for males and 0.29 for females. There was no significant change in rates over time, nor any consistent pattern of risk by region of residence. Birth cohort analysis of mortality showed in each sex, lowest risk for persons born during the Second World War. The age distribution of the tumour was unusual: a progressive rise in both incidence and mortality rates occurred in each sex at ages up to 60-69, at which there was a striking peak, more marked for males and for incidence data, with a sharp decline thereafter. Immigrants from China and Cyprus had significantly high proportional registration ratios, but based on small numbers.

The thymus gland plays an important role in the maturation of immunocompetent T-lymphocyte cells at younger ages. It enlarges until late puberty and then begins a progressive morphological and functional involution (Roitt *et al.*, 1985).

Thymus neoplasms are rare, but since the observation in 1901 that they were associated with myasthenia gravis (Weigart, 1901), there has been interest in their study. Their aetiology remains obscure, however, and their epidemiology is unclear because no population-based study has been carried out (Large *et al.*, 1986); most of the available information comes from case-reports or relatively small hospital series (Wilkins *et al.*, 1966; Bernatz *et al.*, 1973; Salyer & Eggleston, 1976; Maggi *et al.*, 1986). Recently, some clinical studies have suggested that thymus carcinoma may be associated with Epstein-Barr virus (EBV) (Leyvraz *et al.*, 1985; Dimery *et al.*, 1988).

The England and Wales national cancer registry holds an exceptionally large data set from a catchment population of about 50 million, which gave an opportunity to investigate the descriptive epidemiology of these rare tumours and potentially to give clues for the development of specific aetiological hypotheses.

### Materials and methods

### *Sources of data*

Cancer registration has existed at a national level in England and Wales since 1945, and since 1962 it has had complete geographical national coverage. Registration is carried out by regional registries which send data to the national registry at the Office of Population Censuses and Surveys (OPCS) (before 1971 the General Register Office) who collate, analyse and publish the data. Notification of cancers is voluntary and the methods of data collection are not entirely uniform among registries; the indications are that completeness is 95% or better in some regions, varies across the country, and has generally been improving over time (Swerdlow, 1986).

Site of primary malignancy has been coded in the OPCS files according to the Seventh Revision of the 'International Classification of Diseases' (ICD), (World Health Organization, 1957) for 1959-1967 data, the Eighth Revision (World Health Organization, 1967) for 1968-1978, and the Ninth Revision (World Health Organization, 1977) for data from 1979 onwards. Additional codes enable a division by morphological type: using a two-digit OPCS code for 1963-1970 data, the Manual of Tumour Nomenclature and Coding (MOTNAC) (American Cancer Society, 1968) for 1971-1978 data and the International Classification of Diseases for Oncology (ICD-O) (World Health Organization, 1976) for data from 1979 onwards. If no histological information is available, cancers are registered on the basis of clinical diagnosis alone. We extracted from the files of the national registry data on all registrations incident 1963-83 which were coded to thymus cancer (ICD7:195.2; ICD8:194.2; ICD9:164.0). We also extracted, from OPCS mortality files, unpublished data on mortality from thymus cancer 1959-86. Mid-year population estimates for each year of the study period were taken from published sources (General Register Office, 1959-1970; Office of Population Censuses and Surveys, 1971-1986).

### *Methods of analysis*

Directly age-standardised incidence and mortality rates were calculated using England and Wales mid-year populations as denominators and the 'World Standard' population as the standard (Smith, 1987). To reduce random variation when examining secular trends, time series data were smoothed by a moving average of span 5 and weights 1,4,6,4,1/16 (Box & Jenkins, 1970). The figure derived from this smoothing necessarily does not contain separate data points for the two first and two last years.

Models based on the assumption that the observed number of cancer occurrences or cancer deaths arose from a Poisson distribution were fitted to test the statistical significance of linear trends in incidence and mortality (Breslow & Day, 1987), using the GLIM (Generalised Linear Interactive Modelling) (Baker & Nelder, 1978) computer package.

Birth cohort analyses was conducted for mortality but not for incidence, for which insufficient data were available for satisfactory analysis. Standardised cohort mortality ratios (SCMRs) were calculated for each 5-year birth cohort (Case, 1956; Beral, 1974) using, in each sex, the average age-specific mortality of England and Wales 1960-84 as the standard. The significance of SCMRs was tested using the significance factors for the ratio of a Poisson variable to its expectation given by Bailar and Ederer (1964).

Geographical variation in thymus cancer incidence was examined by calculating age-standardised registration rates for hospital regions of residence for 1974-83. This period was chosen because regional boundaries were changed in 1974. Region of residence was known for all registrations in the file.

Age-standardised proportional registration ratios (PRRs) by country of birth were calculated for 1971-83 (PRR of thymus cancer registrations = 100). Birthplace was known for

Correspondence: I. dos Santos Silva.
Received 18 September 1989; and in revised form 25 January 1990.

72% of thymus cancer registration and, of these, 96% were born in the United Kingdom.

The significance of regional rates compared to England and Wales rates, and of proportional registration ratios by country of birth, were tested using the tables of Bailar and Ederer (1964).

### Results

A total of 781 cases of thymus cancer were registered in residents of England and Wales during the 21 year period from 1963 to 1983, of which 54.3% (424) were in males and 45.7% (357) in females.

The majority of the tumours were thymomas (75.4%) (Table I); 16.3% were 'thymus carcinomas'-a heterogeneous group of epithelial neoplasms. Germ cell neoplasms and carcinoid tumours were uncommon. The 'other tumours' category in the table included sarcomas, liposarcomas, vascular and granular tumours. The distribution of histologies was similar in both sexes.

A total of 547 deaths with the underlying cause thymus cancer occurred during 1959–86, of which 51.6% (282) were males and 48.4% (265) were females.

Age-standardised incidence (registration) rates are shown in Figure 1. There was no significant linear trend (males: observed slope $b = -0.007$, $P > 0.10$; females: $b = -0.013$, $P > 0.10$) nor was there any correlation between male and female rates in the same years ($r = 0.05$, $P = 0.83$).

Similarly, annual age-standardised death rates (Figure 1) showed no evidence of a trend over time (males: $b = -0.0005$, $P > 0.10$; females: $b = 0.011$; $P > 0.10$), and also no correlation between male and female death rates for the same years ($r = 0.13$, $P = 0.52$). On examination of mortality by birth cohort (Figure 2), there was no consistent trend, but in each sex, persons born during the Second World War had the lowest risk, although only significantly below 100 for males (males: SCMR = 15, $P < 0.05$; females: SCMR = 59, $P > 0.05$). Both values were based on small numbers of deaths (males = 2; females = 6).

Examining the age distribution of thymus cancer using aggregated data for the entire study period (Figure 3), both incidence and mortality in each sex showed a steady increase in rates up to a peak at ages 60–69, and thereafter a striking decline in rates, particularly in males and for incidence. The overall male excess of thymus cancer incidence was accounted for by the male predominance at the older ages at which the peak occurred, whereas there was no clear sex difference at younger age-groups.

Table II shows the geographical distribution of thymus cancer in England and Wales for 1974–83. Males rates were higher than female rates for all regions except Wessex and Oxford; highest rates in males were in East Anglia followed by the Northern and North East Thames regions. Females rates were highest in Wessex (the region with the lowest male rate), followed by Oxford and East Anglia. There was no correlation between male and female rates for the same regions ($r = 0.25$; $P = 0.363$).

Analysis by country by birth showed significant PRRs for migrants born in China (PRR = 1,852; $P < 0.05$) and Cyprus (PRR = 744; $P < 0.05$), but based on very small numbers of registrations (China = 2; Cyprus = 3).

**Table I** Thymus cancer: histological distribution, England and Wales, 1963–1983

| *Histological type (ICD-0)* | *Number* | *Frequency (%)* |
|---|---|---|
| Thymomas (M8580) | 589 | 75.4 |
| Thymus carcinoma (M8010-M8043) | 127 | 16.3 |
| Germ cell tumours (M9061-M9083) | 15 | 1.9 |
| Carcinoid tumours (M8240) | 2 | 0.3 |
| Other malignancies | 9 | 1.1 |
| Unknown histology (M8003) | 39 | 5.0 |
| Total | 781 | 100.0 |

**Figure 1** Secular trends in thymus cancer incidence 1963–83, and mortality 1959–86, England and Wales (smoothed annual age-standardised rates). Registration: ■, males; *, females. Mortality: +, males; □, females.

**Figure 2** Birth cohort mortality trends from thymus cancer among persons born 1900–1960, England and Wales. ■, males; +, females.

**Figure 3** Age distribution of thymus cancer in England and Wales. Mean annual registration rates 1963–83 and mortality rates 1959–86. Symbols as Figure 1.

### Discussion

Some reservations regarding diagnosis of thymus cancer in the present data need to be considered. First, thymomas are considered as malignant because of local invasion or distant metastases (Rosai, 1985), since the vast majority are morphologically indistinguishable from benign thymomas (Chen, 1984). The distinction between slight invasion and no invasion is not always clear, however (Jain & Frable, 1974). Despite these uncertainties, the similarity of the results for incidence with those for mortality in the present study give some indication that such diagnostic effects are unlikely to have affected the quality of the incidence data greatly. Second, even to establish that a tumour has originated from the thymus parenchyma is not always an easy task. For instance the nature of 'granulomatous thymomas' and 'seminomatous thymomas', and their relation to Hodgkin's disease (Keller & Castleman, 1974) and germ cell tumours (Levine, 1973) respectively, remains unclear. Since they are a very small proportion of thymus cancer, however, their misdiagnosis would have had a negligible impact in our data.

**Table II** Incidence of thymus cancer by hospital region of residence, England and Wales, 1974–83

| | *Males* | | *Females* | |
|---|---|---|---|---|
| *Region* | *No. 1974–83* | *Mean annual registration rate*[a] | *No. 1974–83* | *Mean annual registration rate*[a] |
| Northern | 18 | 1.01 | 12 | 0.63 |
| Yorkshire | 17 | 0.83 | 11 | 0.49 |
| Trent | 15 | 0.52 | 14 | 0.41 |
| East Anglia | 14 | 1.31[b] | 8 | 0.89 |
| N.W. Thames | 16 | 0.78 | 11 | 0.49 |
| N.E. Thames | 19 | 0.89 | 14 | 0.62 |
| S.E. Thames | 13 | 0.64 | 6 | 0.28 |
| S.W. Thames | 9 | 0.51 | 7 | 0.36 |
| Wessex | 5 | 0.28[b] | 15 | 0.92 |
| Oxford | 8 | 0.60 | 12 | 0.91 |
| S. Western | 17 | 0.87 | 8 | 0.52 |
| W. Midlands | 20 | 0.64 | 12 | 0.44 |
| Mersey | 3 | 0.44 | 6 | 0.42 |
| N. Western | 15 | 0.65 | 12 | 0.42 |
| Wales | 8 | 0.52 | 7 | 0.41 |
| England and Wales | 197 | 0.68 | 155 | 0.52 |

[a]Rate per million directly age-standardised to the 'world' population (Smith, 1987).
[b]Differs significantly from England and Wales at $P<0.05$.

Two additional issues of coding should also be noted. First, thymomas might fail to be coded as thymus cancer if the word malignant was arbitrarily omitted on the medical record. Second, the thymus cancer data exclude thymic lymphomas which are allocated in the ICD to lymphoma rather than thymus cancer. The impact of the first is difficult to assess. The effect of the second would have been small since lymphomas arising primarily within the thymus gland are rare (Keller & Castleman, 1974).

That mortality rates were substantially lower than the corresponding incidence rates agree with clinical studies which report a relatively good survival for malignant thymomas, which form the great majority of thymus cancers (Batata *et al.*, 1974; Verley & Hollman, 1985; Maggi *et al.*, 1986). Since classification might be ambiguous, no attempt was made to analyse the data further by histological category.

Some clinical reports have suggested an increasing frequency of diagnosis of thymomas in the past decade (Maggi *et al.*, 1986), but considered that this was probably due to changes in diagnostic and therapeutic approaches for myasthenia gravis rather then a true increase in incidence. Our population-based study did not show an increase in incidence. Since diagnostic or therapeutic policy changes in the recent period may have led to the detection of small thymomas which would not have been detected in other ways, and cancer registration has probably improved over time (Swerdlow, 1986), our data might hide a possible decrease in incidence over time but are unlikely to correspond to an increase. The lack of correlation between male and female rates over the regions of England and Wales suggests that our incidence data have not been grossly affected by regional incompleteness, which would presumably have affected both sexes similarly. A lack of secular increase also corresponds with data from clinical series from Papworth Hospital, Cambridge (Large *et al.*, 1986).

The age distribution of thymus cancer is unusual. Lymphoreticular cancers (Greene, 1982), nasopharyngeal cancer (NPC) (Shanmugaratnam, 1982) and certain other tumours arising in immunodeficient conditions for which a viral aetiology has been suspected (Kinlen, 1982), share with thymus cancer a peak in late middle age, but show some differences in age distribution, notably in the degree of decrease at older ages. The distribution of thymus cancer is particularly similar to that of NPC in high incidence populations, e.g. Singapore (Shanmugaratnam, 1982), and also of Creutzfeldt-Jakob disease (P.G. Smith, personal communication), a neurological disorder suspected of being caused by a slow viral agent (Roos *et al.*, 1973). In the cancers above, rates start to rise earlier and steeper than in most malignancies, which may suggest that exposure to carcinogenic agents begins very early in life. The extraordinary decline of thymus cancer after late middle age might perhaps indicate cessation of exposure to an aetiological agent or a decreasing number of susceptibles in the population, although in part it might be due to the decline at older ages in thymectomies for myasthenia and, hence, less probability of detecting clinically silent thymomas. Rates start to go down when the thymus gland is already morphologically involuted but still preserves some functional activity (Roitt *et al.*, 1985).

The similarities with NPC are particularly interesting because the nasopharynx and thymus have the same embryological origin from the primitive foregut (Leyvraz *et al.*, 1985), and because a serological association with EBV has been well documented for NPC (Shanmugaratnam, 1982) and has recently been reported for thymus cancer (Leyvraz *et al.*, 1985; Dimery *et al.*, 1988). Like thymus cancer, NPC also shows stable time trends (Shanmugaratnam, 1982). The most outstanding epidemiological feature of NPC, however, is its very high incidence in southern Chinese, both in and outside China (Shanmugaratnam, 1982). High rates are found in other groups in South East Asia, especially Filipinos, and in Tunisians (Hirayama, 1978). Although our data showed a significant high risk of thymus cancer among persons born in China and in Cyprus, no coherent pattern was present. Thymus cancer data have only been published in *Cancer Incidence in Five Continents* (Muir *et al.*, 1987) in the latest edition, and very small numbers make interpretation difficult: apart from a very high incidence (1.7 per 100,000 per annum) in Filipino males in Bay Area, United States, no clear geographical or ethnic differentials were present.

The low SCMRs observed in both sexes for the generation born during the Second World War might also relate to a putative viral aetiology, since a small average family size characterised that time (Beral *et al.*, 1978) and, hence, perhaps there was a lesser probability of acquiring infectious diseases (Reves, 1985). The method used to calculate the cohort ratios tend to underestimate real decreases, because of the overlap of adjacent generations (Beral, 1974) and because of the inevitable use of the overall data to generate the expected values. It should be noted also that the ratios were based on small numbers. Since these are recent birth cohorts, their follow-up as they age will help to clarify this issue.

The role of EBV in human cancers is still controversial even for NPC, a tumour with which the virus has been strongly associated (Henderson, 1989). That thymic cells may contain EBV genome has been postulated in reports of EBV-induced malignant lymphoproliferative disorders arising in patients subject to thymic transplantation (Reece *et al.*, 1981), but the relation, if any, with thymus cancer is unclear.

So far, EBV has only been shown to be associated with lymphoepitheliomas (Leyvraz *et al.*, 1985; Dimery *et al.*, 1985), a small sub-group of thymus carcinoma (Chen, 1984), and it is not known if there is a link for other morphological types of thymus cancer.

Further epidemiological investigation of the parallels between NPC and thymus cancer may be worthwhile to pursue.

This work was partly developed by I. Silva as part of her MSc dissertation at the London School of Hygiene and Tropical Medicine in 1988. We thank Junta National de Investigação Científica e Tecnológica (JNIC), Lisbon, for support of I. Silva during her MSc course, and Mrs T. Buckett for help in the extraction of data.

### References

AMERICAN CANCER SOCIETY (1968). *Manual of Tumour Nomenclature and Coding*. American Cancer Society: New York.

BAILAR, J.C. III & EDERER, F. (1964). Significance factors for the ratio of a Poisson variable to its expectation. *Biometrics*, **20**, 639.

BAKER, R.J. & NELDER, J.A. (1978). *GLIM-Generalised Linear Interactive Modelling*. Royal Statistical Society: London.

BATATA, M.A., MARTINI, N., HUVOS, A.G., AGUILLAR, R.I & BEATTIE, E.J. (1974). Thymomas: clinicopathological features, therapy, and prognosis. *Cancer*, **34**, 389.

BERAL, V. (1974). Cancer of the cervix: a sexually transmitted infection? *Lancet*, **i**, 1037.

BERAL, V., FRASER, P. & CHILVERS, C. (1978). Does pregnancy protect against ovarian cancer? *Lancet*, **i**, 1083.

BERNATZ, P.E., KHONSARI, S., HARRISON, E.G. & TAYLOR, W.F. (1973). Thymoma: factors influencing prognosis. *Surg. Clin. North Am.*, **53**, 885.

BOX, E.P. & JENKINS, G.M. (1970). *Time Series Analysis Forecasting and Control*, p. 10. Holden-Day: London.

BRESLOW, N.E. & DAY, N.E. (1987). *Statistical Methods in Cancer Research, Vol.II. The Design and Analysis of Cohort Studies*, p. 136. International Agency for Research on Cancer: Lyon.

CASE, R.A.M. (1956). Cohort analysis of mortality rates as an historical or narrative technique. *Br. J. Prev. Soc. Med.*, **10**, 1959.

CHEN, K.T.K. (1984). Squamous carcinoma of the thymus. *J. Surg. Oncol.*, **25**, 61.

DIMERY, I.W., LEE, J.S., BLICK, M., PEARSON, G., SPITZER, G. & HONG, W.K. (1988). Association of the Epstein-Barr virus with lympoepithelioma of the thymus. *Cancer*, **61**, 2475.

GENERAL REGISTER OFFICE (1959-70). *The Registrar General's Statistical Review of England and Wales*. HMSO: London.

GREENE, M.H. (1982). Non-Hodgkin's Lymphoma and Mycosis Fungoides. In *Cancer Epidemiology and Prevention*, Schottenfeld, D. & Fraumeni, J.F. (eds) p. 754. W.B. Saunders: Philadelphia.

HENDERSON, B.E. (1989). Establishment of an association between a virus and a human cancer. *J. Natl Cancer Inst.*, **81**, 31.

HIRAYAMA, T. (1978). Descriptive and analytical epidemiology of nasopharyngeal cancer. In *Nasopharyngeal Carcinoma: Etiology and Control*, de-Thé, G., Ito, Y. & Davis, W. (eds) p. 167. IARC: Lyon.

JAIN, U. & FRABLE, W.J. (1974). Thymoma. Analysis of benign and malignant criteria. *J. Thorac. Cardiovasc. Surg.*, **67**, 310.

KELLER. A.J. & CASTLEMAN, B. (1974). Hodgkin's disease of the thymus gland. *Cancer*, **33**, 1615.

KINLEN, L.J. (1982). Immunologic Factors. In *Cancer Epidemiology and Prevention*, Schottenfeld, D. & Fraumeni, J.F. (eds) p. 494. W.B. Saunders: Philadelphia.

LARGE, S.R., SHNEERSON, J.M., STOVIN, P.G. & WALLWORK, J. (1986). Surgical pathology of the thymus: 20 years' experience. *Thorax*, **41**, 51.

LEVINE, G.D. (1973). Primary thymic seminoma–a neoplasm ultrastructurally similar to testicular seminoma and distinct from epithelial thymoma. *Cancer*, **31**, 729.

LEYVRAZ, S., HENLE, W., CHAHINIAN, A.P. & 5 others (1985). Association of Epstein-Barr virus with thymic carcinoma. *N. Engl. J. Med.*, **312**, 1296.

MAGGI, G., GIACCONE, G., DONADIO, M. & 8 others (1986). A review of 169 cases, with particular reference to results of surgical treatment. *Cancer*, **58**, 765.

MUIR, C., WATERHOUSE, J., MACK, T., POWELL, J. & WHELAN, S. (eds) (1987). *Cancer Incidence in Five Continents, Vol. V*, p. 814. International Agency for Research on Cancer: Lyon.

OFFICE OF POPULATION AND CENSUSES AND SURVEYS (1971-86). *Cancer Statistic Registrations*. Series MB1, nos 1,2,4,5,7,8,10-16. London: HMSO.

REECE, E.R., GARTNER, J.G., SEEMAYER, T.A., JONCAS, J.H. & PAGANO, J.S. (1981). Epstein-Barr virus in a malignant lymphoproliferative disorder of B-cells occurring after thymic epithelial transplantation for combined immunodeficiency. *Cancer Res.*, **41**, 4243.

REVES, R. (1985). Declining fertility in England and Wales as a major cause of the twentieth century decline in mortality. *Am. J. Epidemiol.*, **122**, 112.

ROITT, I., BROSTOFF, J. & MALE, D. (1985). *Immunology*. Gower Medical Publishing: London.

ROOS, R., GAJDUSEK, D.C. & GIBBS, C.J. Jr (1973). The clinical characteristics of transmissible Creutzfeldt-Jakob disease. *Brain*, **96**, 1.

ROSAI, J. (1985). 'Lymphoepithelioma-like' thymic carcinoma: another tumour related to Epstein-Barr virus? *N. Engl. J. Med.*, **312**, 1320.

SALYER, W.R. & EGGLESTON, J.C. (1976). Thymoma: a clinical and pathological study of 65 cases. *Cancer*, **37**, 229.

SHANMUGARATNAM, K. (1982). Nasopharynx. In *Cancer Epidemiology and Prevention*, Schottenfeld, D. & Fraumeni, J.F. (eds) p. 536. W.B. Saunders: Philadelphia.

SMITH, P.G. (1987). Comparisons between registries: age-standardized rates. In *Cancer Incidence in Five Continents, Vol. V*. Muir, C., Waterhouse, J., Mack, T., Powell, J. & Whelan, S. (eds) p. 671. International Agency for Research on Cancer: Lyon.

SWERDLOW, A.J. (1986). Cancer registration in England and Wales: some aspects relevant to interpretation of the data. *J.R. Stat. Soc.*, **149**, 146.

VERLEY, J.M. & HOLMANN, K.H. (1985). Thymoma: A comparative study of clinical stages, histologic features, and survival in 200 cases. *Cancer*, **55**, 1074.

WEIGART, C. (1901). Pathologisch-anatomischer Beitrag zur Erb'schen Krankheit (Myasthenia gravis). *Neurol. Zbl.*, **20**, 597.

WILKINS, E.W., EDMUNDS, L.H. & CASTLEMAN, B. (1966). Cases of thymoma at the Massachusetts General Hospital. *J. Thorac. Cardiovasc. Surg.*, **52**, 322.

WORLD HEALTH ORGANIZATION (1957, 1967, 1977). *Manual of the International Statistical Classification of Diseases, Injuries, and Causes of Death*, Seventh Revision, Eighth Revision, Ninth Revision. World Health Organization: Geneva.

WORLD HEALTH ORGANIZATION (1976). *International Classification of Disease for Oncology*. World Health Organization: Geneva.